# Plant-growth-promoting rhizobacteria for mitigating salinity stress in rice farming: a review of the Vietnamese Mekong Delta

**DOI:** 10.3389/fpls.2025.1635193

**Published:** 2025-10-03

**Authors:** Trinh Thi My Nguyen, Dung Minh Ha-Tran, Chieh-Chen Huang

**Affiliations:** ^1^ Faculty of Creative Technology, School of Technology, Van Lang University, Ho Chi Minh City, Vietnam; ^2^ Laboratory of Ecology and Environmental Management, Science and Technology Advanced Institute, Van Lang University, Ho Chi Minh City, Vietnam; ^3^ Faculty of Applied Technology, School of Technology, Van Lang University, Ho Chi Minh City, Vietnam; ^4^ Department of Life Science, National Chung Hsing University, Taichung, Taiwan; ^5^ Advanced Plant and Food Crop Biotechnology Center, National Chung Hsing University, Taichung, Taiwan; ^6^ Innovation and Development Center of Sustainable Agriculture, National Chung Hsing University, Taichung, Taiwan

**Keywords:** salinity intrusion, plant growth promoting rhizobacteria, salt stress tolerance, rice production, Vietnamese Mekong Delta

## Abstract

Salinity intrusion, exacerbated by climate change and anthropogenic activities, poses a significant global threat to agricultural productivity, particularly in coastal and deltaic regions. Rice, a staple crop critical for food security and economic stability in many developing nations, is highly susceptible to salt stress, which reduces yields and threatens livelihoods. In the Vietnamese Mekong Delta (VMD), a key rice-producing region, recurrent drought-induced salinity events have caused substantial damage to agriculture, and the economic well-being of millions of residents. These events highlight the urgent need for sustainable solutions to maintain rice production under adverse environmental conditions. Plant-Growth-Promoting Rhizobacteria (PGPR) have emerged as a promising eco-friendly approach to enhance plant salt tolerance, offering potential to mitigate salinity stress in rice crops. Here we review the role of PGPR in alleviating salinity stress in rice farming in the VMD, highlighting its potential as a sustainable agricultural approach. The review synthesizes existing research to assess the causes of salinity intrusion, the efficacy of PGPR, and the limitations of current studies in this region. The major points are the following: 1) Saline intrusion in the VMD is driven by multiple factors, including sea-level rise, land subsidence, upstream dams’ operation, and excessive sand mining, which exacerbate agricultural challenges; 2) PGPR enhance rice salt tolerance through mechanisms such as osmotic regulation, improved nutrient uptake, and activation of stress-responsive genes, as evidenced in controlled and field studies; 3) Research in Vietnam is constrained by a lack of long-term investigations and a reliance on publications in Vietnamese-language scientific journals, which may limit international attention and rigorous peer-review processes, necessitating further studies to support scalability and adoption by VMD farmers, and also enlarge international collaboration in this important field of study.

## 1 Introduction

Rice is a staple food for billions of peoples worldwide, especially in Asia (Vinci et al., 2023). Rice production and exportation are critical to Vietnam’s food security and economy (Maitah et al., 2020). In 2023, Vietnam ranked as the third-largest rice exporter globally, following India and Thailand. The agricultural regions surrounding the Mekong River are renowned for their fertile soil and biodiversity, making them ideal for rice and other crop cultivation. However, these regions are increasingly threatened by climate change, notably sea-level rise. Vietnam is one of five countries, along with China, Japan, India, and Bangladesh, most severely affected by sea-level rise. The Red River Delta and Mekong River Delta, two Vietnam’s primary rice production hubs, face significant risks from this environmental challenge. Based on some previsions, a one-meter rise of seawater may lead to the submergence of 0,3–0,5 million hectares (ha) of the Red River Delta and approximately 90% of the Vietnamese Mekong Delta (VMD) (Danh and Khai, 2014). In addition, land subsidence in the VMD poses an even greater threat than sea-level rise, as the rate of land sinking surpasses that of rising sea levels (Dunn and Minderhoud, 2022). Salinity intrusion in the VMD is currently a critical environmental challenge caused by a combination of climate change and anthropogenic factors. The rise of seawater damages coastal areas in this region and upstream damming of the Mekong River reduces freshwater flow, especially during the dry season, thereby diminishing the natural barrier against saltwater intrusion (Tri, 2019; Van Tho, 2022). Additionally, excessive groundwater extraction for agriculture, aquaculture, industry, and domestic activities accelerates land subsidence by compacting the underlying soil layers, which results in land sinking (Van Tho, 2022). This unsustainable practice amplifies the extent of saline intrusion. These factors have profound impacts on arable land, thus significantly reducing rice yields (Binh et al., 2025). Soil degradation, shortage of freshwater and declined agricultural productivity threaten national food security and livelihoods of millions of farmers in the VMD, necessitating appropriate solutions to remain rice production stability in this prominent region.

Most rice varieties are glycophytic, so their growth and yield can be severely impacted by elevated salt concentrations (Hoang et al., 2016). Rice is the most salinity-sensitive cereal crop, with an electrical conductivity (EC) of 3 dS m^−1^, even below the threshold for generally recognized saline soil, causing a 10% loss of yield for most cultivated varieties (Hoang et al., 2016). In regions like the VMD, where conditions are challenging, ensuring stable rice yields and farmer’s income is a priority for breeding programs and agricultural technology advancements. The rising sea levels in the VMD present significant challenges for local farmers, offering critical opportunities to study their adaptive responses to a harsh environment. So far, numerous plant breeding programs are focused on developing rice varieties with multiple tolerant traits to abiotic stresses, including salinity, flooding, and water deficits (Crop Trust, 2021). Despite various approaches to enhance salinity tolerance in rice, significant breakthroughs remain elusive. Developing salt-tolerant rice through conventional breeding or biotechnology is labor-intensive and time-consuming. Moreover, challenges such as limited parental resources, complex genomic traits (e.g., multiple genes involved in salinity tolerance), and transgene silencing continue to impede progress in variety improvement (Qin et al., 2020).

Among various strategies to enhance salt stress tolerance in rice, the implementation of plant growth-promoting rhizobacteria (PGPR) offers an environmentally sustainable and effective approach. However, in Vietnam, research on the effects of PGPR on the rice plant growth, development, nutrient uptake, phosphate (PO4^3−^) and silicate (Si) solubilization, nitrogen (N) fixation, and mitigation of salinity’s adverse impacts remain limited (Habibi et al., 2014; Hussain et al., 2022). By far, finding PGPR-related studies published in Scopus- or ISI-indexed journals is challenging, with few available (Rose et al., 2014; Khuong et al., 2021). Based on our findings, the 15-year study on the biofertilizer BioGro by Nguyen et al. (2017) is the only long-term investigation conducted in Vietnam to date (Nguyen et al., 2017), while most other papers published in Vietnamese-language scientific journals (Đường et al., 2018; Đường and Nghĩa, 2020; Chiêu and Hiệp, 2010; Giang et al., 2024; Huy and Hiệp, 2019). This knowledge gap and limited accessibility hinders advancements in PGPR engineering, commercialization, as well as international collaboration. In this work, we evaluate the current state of saline intrusion in the VMD, highlight salient findings from PGPR studies in Vietnam, and explore the potential use of indigenous strains to enhance rice tolerance to salt stress in Vietnam.

## 2 Key drivers of salinity intrusion

### 2.1 Climatic and geological factors

The VMD, spanning 39,000 km^2^, is Vietnam’s primary rice production region, which has contributed over 50% of national rice yield and 90% of rice exports (Thach et al., 2023; Tuan et al., 2024). However, saline intrusion, driven by complex interactions of climatic, geological, and anthropogenic factors, poses significant threat to this agricultural region. Coastal provinces, such as Ben Tre, Tra Vinh, Soc Trang, and Ca Mau, confront heightened vulnerability due to their inherently low elevation and proximity to the East Sea. Since the 1990s, salinity intrusion has increased its magnitude, penetrating deeper inland and lasting longer, particularly during the dry season. This phenomenon disrupts irrigation systems which are critical for rice cultivation. Salinity intrusion jeopardizes farmer livelihoods and necessitate innovative solutions like salt-tolerant rice varieties and plant growth promoting rhizobacteria (PGPR). The primary causes and effects of saline intrusion are summarized in **Table 1**.

**Table 1:** Causes and effects of saline intrusion in the VMD.

Cause	Mechanism	Impacts on salinity intrusion	Effect on rice production and economy	References
Sea-level rise	Facilitates upstream seawater movement, increase tidal influence on lowland regions	Early onset, deeper inland penetration, longer duration of intrusion	Reduces arable field; yield losses of 2,5–4 ton ha^−1^; economic losses up to 337 million USD	(Danh and Khai, 2014; Kantoush et al., 2017; Park et al., 2022; Toan, 2014)
Land subsidence	Groundwater extraction lowers elevation, enhancing saltwater intrusion	Amplifies negative effects of sea-level rise, weakens infrastructure protection	Degrades soil quality; reduces rice yields and farmland availability	(Erban et al., 2014; Minderhoud et al., 2017)
Upstream dams	Reduces freshwater flow and sediment load, increases salinity in irrigation system during dry season	Increases saline water penetration, weakens freshwater dilution capacity	Lowers rice productivity; contributes to drought and economic losses	(Loc et al., 2021; Phuong et al., 2018)
Riverbed sand mining	Deepens riverbeds, alters river morphology, destabilizes irrigation systems	Enhances tidal influences and river incision, allowing saltwater intrusion	Reduces rice yields; increases irrigation challenges and economic losses	(Binh et al., 2020; Hackney et al., 2020)

Sea-level rise, which is propelled by climate change, accelerates saline intrusion by penetrating seawater into the irrigation canal network in the VMD. Previous data indicate that salinity intrusion has worsened since the 1990s, and projections suggest a one-meter sea-level rise could submerge approximately 90% of the VMD by 2100, detrimentally affecting provinces like Bac Lieu (39% land at risk) and Tra Vinh (Danh and Khai, 2014; Ngo et al., 2016). During the dry season, low river discharge and tidal amplification enable seawater to penetrate 50–130 km inland, which salinizes irrigation systems in Ben Tre and Soc Trang, where rice fields heavily rely on freshwater (Nguyen et al., 2024).

### 2.2 Anthropogenic factors

Upstream hydropower dams in the Mekong River Basin significantly alters downstream hydrology by reducing freshwater flow, intensifying salinity intrusion during the dry season. By 2016, 56 dams, including mega-dams Xiaowan and Nuozhadu, disrupt flow patterns, decrease frequency and intensity of seasonal floods, contributing to severe droughts in 2015–2016 and 2019–2020 (Phuong et al., 2018). These upstream dams also trap nutrient-rich sediments, reducing soil fertility and exacerbating salinity destructive impacts (Loc et al., 2021). The limited coordination and collaboration restrict Vietnam to secure sufficient amount of freshwater for irrigation and household consumption, especially during dry season. Furthermore, riverbed sand mining with approximately 8,5–45,7 Mm^3^ annually causes riverbed incision, pushing seawater intrusion and destabilizing riverbanks (Hackney et al., 2020). Illegal sand mining, a long lasting and unsolvable environmental issue, which is linked to construction demand, magnifies ecological damage, including loss of fish habitats that support rice-based agroecosystems (Park et al., 2020; Yuen et al., 2024) (Park et al., 2020; Yuen et al., 2024). Additionally, land subsidence, which fundamentally caused by excessive extraction of groundwater for domestic, agricultural, and industrial activities, aggravates this fragility. Subsidence rates average around 2,5 cm year^−1^ and reach 4 cm year^−1^ in Ca Mau, outpacing global sea-level rise (2,8–3,6 mm year ^−1^). This crucial threat lowers the VMD’s elevation and increases flooding risks in rice field (Minderhoud et al., 2017; Qin et al., 2013). Moreover, over-extraction of groundwater has depleted aquifers, leading to the infiltration of saline water through capillary rise, degrading arable land (Erban et al., 2014).

### 2.3 Effects on rice production and economy

**Figure 1:**
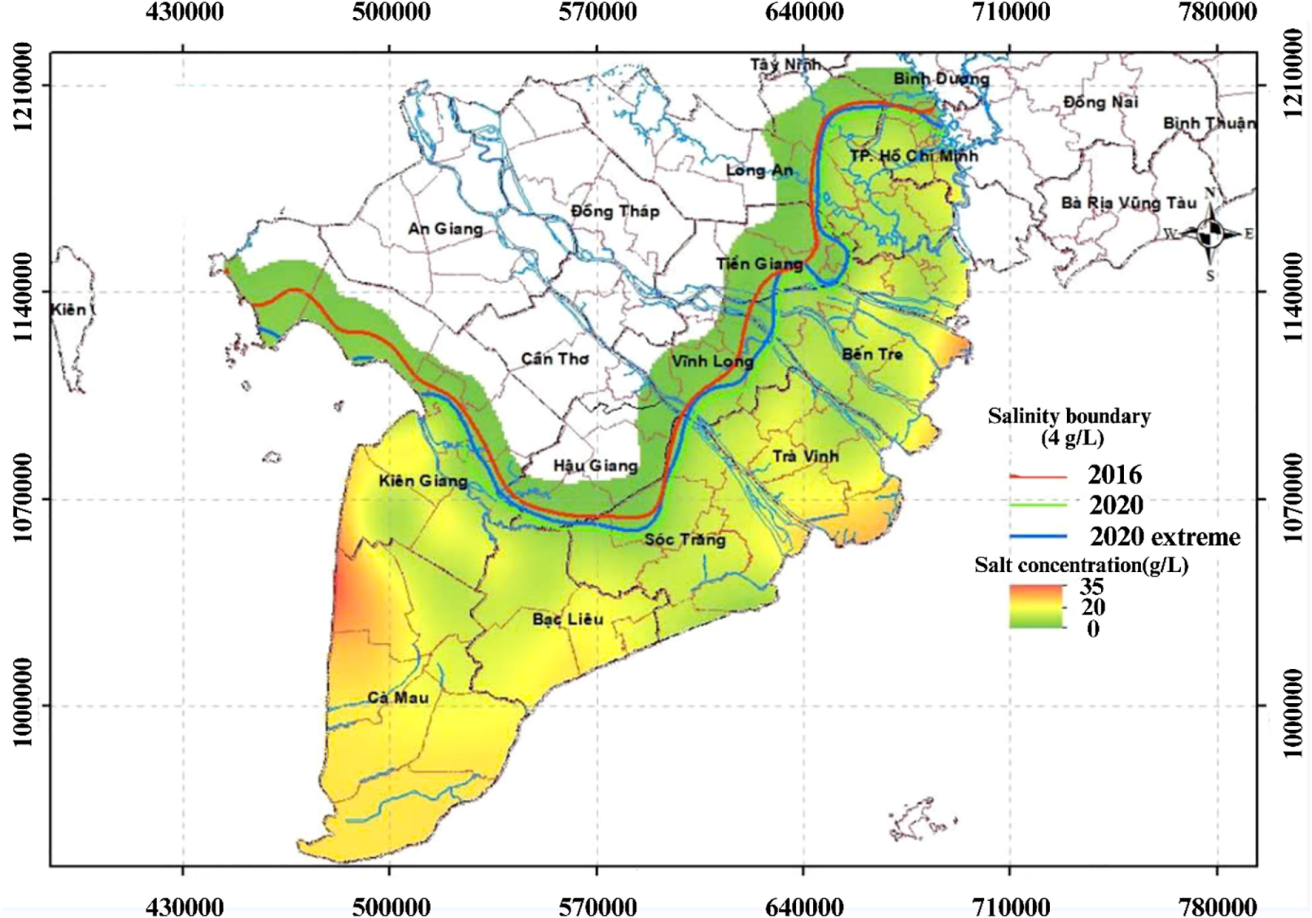
Salinity intrusion in the VMD in 2016 and 2020. Map of salinity intrusion in the Vietnamese Mekong Delta, showing salinity boundaries (4 g L^−1^) for 2016 (red), 2020 (green), and 2020 extreme scenario (blue), with salt concentration distribution (0–35 g L^−1^) indicated by a color gradient. The map highlights the spatial extent of saltwater intrusion along major rivers and coastal provinces, reflecting changes over time. Source: (Central Steering Committee for Natural Disaster Prevention and Control (CCNDPC), 2020).

Saline intrusion significantly damages rice production. During severe events such as the El Nino-driven droughts and reduced river flow in 2015–2016 and 2019–2020, the impacts were extensive (**Figure 1**). In 2016, approximately 224,000 ha of paddy field, 13,000 ha of cash crops, 25,500 ha of fruit trees, and 14,400 ha of aquaculture were damage (CGIAR Research Centers in Southeast Asia, 2016). In the 2020 event, salt concentrations reached 4 g L^−1^, with seawater penetrating 50–130 km into major rivers, affecting an estimated 215,445 ha of rice and causing economic losses of 337 million USD (Park et al., 2022; Baca et al., 2017). Household affected by salinity intrusion experience lower total production with approximately 761,47 kg ha^−1^ less for rice, resulting in reduced total and net revenues compared to unaffected farmers (Thanh et al., 2023). In Lich Hoi Thuong area, Soc Trang province, salinity reduced rice yields by 2,54 tons ha^−1^ annually (Khai et al., 2018). Besides agriculture, these climatic events also disrupted water supplies for millions of residents in the VMD (Tran and Yong, 2025).

Salinity has driven significant shifts in agricultural practices, moving farmers from traditional triple- or double-rice cropping to aquaculture, or even pushing farmer displacement and migration to urban sectors (Binh et al., 2025; Le et al., 2022; Tran et al., 2021). Adaptation strategies including sluice gates and dikes cost money but also face challenges due to funding restraints and rising salinity levels (Duy et al., 2025; Tran et al., 2021). In the near future, Vietnam faces economic challenges including reduced global rice export competitiveness and increased food security risks (Vietnam Chamber of Commerce and Industry (VCCI), 2025). Therefore, enhanced transboundary water management and an intensified investment in salinity-tolerant rice and PGPR technologies are strongly required to address these pressing issues (Mills et al., 2025; Pdr and Nam, 2017).

## 3 The adverse effects of salinity on rice growth and development

### 3.1 Environmental context and emerging threats

Traditionally, the VMD relied on seasonal flooding to enrich soils and wash out toxic residuals from rice fields, supporting agricultural production. The floodwaters supplied alluvial sediment, rejuvenating the fields with essential macronutrients such as N, phosphorous (P), potassium (K), calcium (Ca), magnesium (Mg), and sulfur (S) and micronutrients such as boron (B), iron (Fe), copper (Cu), manganese (Mn), molybdenum (Mo), and zinc (Zn). The amount of sediment largely varied from few to ten tons per hectare. Interestingly, natural fish in the floodwaters serve as biocontrol agents in rice ecosystems (Tong, 2017). However, in recent decades, many contributors, such as climate change, upstream dam operation, sea-level rise, and reduced freshwater discharge, intensify salinity intrusion. This threat jeopardizes rice cultivation and risks livelihood of million people in the region (Hoang et al., 2018). On a global scale, soil salinization has emerged as a critical constraint on crop production. In some provisions, by 2050, nearly half of the global arable land could be affected by salinity, dramatically reducing food security worldwide (Hasanuzzaman et al., 2014). Salinization can be classified into two types: primary salinization originated from natural processes such as mineral weathering and salt accumulation via capillary rise from saline groundwater, and secondary salinization caused by anthropogenic factors, for example improper irrigation practices and poor drainage (Butcher et al., 2016; Mohanavelu et al., 2021).

### 3.2 Ion imbalances, osmotic stress and plant stress responses

Salinity alters soil chemistry by increasing concentrations of primary cations (Na^+^, K^+^, Ca^2+^, and Mg^2+^), and anions (Cl^−^, SO_4_^2−^, NO_3_^2−^, and HCO_3_^−^). Among them, Na^+^ and Cl^−^ are considered the most harmful contributors to rice metabolism (Corwin, 2021). Excess Na^+^ disrupts soil structure and enzyme activity, while Cl^−^ interfere with photosynthesis. Ion toxicity disrupts intracellular signaling and displaces essential nutrients, particularly K^+^, leading to nutrient deficiency (Assaha et al., 2017). The accessibility of other important nutrients like Ca^2+^, Mg^2+^, Mn^2+^, and Fe^2+^ are also reduced by the abundance of Na^+^ and Cl^−^ in soils (Tavakkoli et al., 2010).

An obvious effect of salinity stress is osmotic stress, which is caused by a rise of soil’s osmotic potential, restricting plant water uptake. This results in dehydration-like symptoms, stomatal closure, and suppressed photosynthesis (Katori et al., 2010). In general, plant responses to salinity stress occur in two phases: (i) an initial, rapid osmotic stress phase, or so-called ion-independent, happening within minutes to days, primarily affecting water uptake and cell turgor. The ion-independent phase involves rapid signaling cascades and hormonal adjustment in response to Na^+^ influx; (ii) a slower, long-term ion toxicity phase, so called ion-dependent response, lasting days to weeks and is characterized by toxic ion accumulation in shoots (Balasubramaniam et al., 2023; Munns and Tester, 2008).

### 3.3 Antioxidant and hormonal responses

The metabolic disruptions lead to an overproduction of reactive oxygen species (ROS) such as superoxide radicals, hydrogen peroxide, and hydroxyl radicals (Khan et al., 2016). Oxidative stress triggers significant cellular damages, including lipid peroxidation in membranes, which indicated by increased malondialdehyde (MDA), and MDA content, and increases electrolyte leakage (Degon et al., 2023; Jaemsaeng et al., 2018). Hormones like abscisic acid and ethylene rise dramatically in response to salt, regulating stomatal behavior and stress signaling. Ethylene biosynthesis, driven by 1-aminocyclopropane-1-carboxylic acid (ACC) oxidase, also leads to the emission of volatile organic compounds (VOCs). These VOCs (e.g., benzenoids, terpenes, and aldehydes) play dual roles as antioxidants and interplant messengers (Chatterjee et al., 2018). An excess amount of ethylene is often associated with senescence (Dubois et al., 2018).

### 3.4 Overall impact on rice growth and yield

Growth and development of rice plants are obviously impacted under salinity conditions. Salinity reduces plant height and leaf expansion, shoot and root biomass, and survival rates. It negatively impacts yield components such as panicle length, thousand-grain weight, percentage of filled grains, and the number of effective tillers (Debapriya Choudhury et al., 2024; Li et al., 2024). Early exposure to salinity during the grain-filling stage has been shown to reduce grain quality and yield in fragrant rice cultivars, with average yield reductions of 33,8% across four consecutive seasons in Thailand (Dangthaisong et al., 2023). Rice variety Huanghuazhan, after exposure to 50 mM sodium chloride (NaCl) for two weeks, showed severe chlorophyll degradation, root inhibition, and reduced biomass (Xue et al., 2024).

## 4 Integrated mechanisms of salt stress tolerance in rice using omics approach

### 4.1 Genes involved in plant response to salinity stress

Rice plants have evolved a complex, multi-layered defense system to cope with the detrimental effects of salt stress. This adaptation involves a tightly coordinated interplay of molecular, physiological, and even biotic mechanisms, from maintaining cellular ion balance to recruiting beneficial microbes in the root environment. Recent studies, which utilized high-throughput omics platforms, has illuminated these integrated pathways, allowing for a comprehensive understanding of how tolerant genotypes thrive under saline conditions. A primary challenge under salt stress is the toxic accumulation of Na^+^ ions. Rice plants manage this through a combination of ion transport and osmotic adjustment (Kumar et al., 2013). The Salt Overly Sensitive (SOS) pathway represents a fundamental line of defense. When high salt levels induce cytosolic Ca^2+^ spikes, the Ca^2+^-binding protein SOS3 senses this signal, activating the kinase SOS2. This, in turn, phosphorylates the plasma membrane Na^+^/H^+^ antiporter SOS1, which actively extrudes Na^+^ from the roots, thus preventing its upward movement to the shoots (Qiu et al., 2002; Xiao and Zhou, 2023). In addition to this extrusion mechanism, rice employs intracellular strategies. The vacuolar Na^+^/H^+^ antiporter OsNHX1 sequesters excess Na^+^ into the vacuole, effectively isolating it from the cytoplasm to maintain cellular ion homeostasis and protect metabolic processes (Fukuda et al., 2004). Similarly, the high-affinity K**^+^** transporter *OsHKT1;5*, located on the Saltol QTL, plays a crucial role in maintaining the essential K^+^ balance (Platten et al., 2013). It facilitates the unloading of Na^+^ from the xylem into root cells, thereby reducing its harmful accumulation in the shoots (Ren et al., 2005). The expression of *OsHKT1;5* is itself meticulously regulated by a complex centered on the transcription factor *OsMYB106* and its cofactors *OsDNAJ15*, *OsSUVH7*, and *OsBAG4* (Liu et al., 2023), highlighting a key intersection between ion transport and transcriptional control. Beyond managing ion toxicity, rice plants also counter the osmotic stress. This is achieved by accumulating compatible solutes like proline and various sugars, which act as osmolytes to maintain turgor pressure. This process is often complemented by robust antioxidant defense mechanisms involving enzymes such as superoxide dismutase (SOD), peroxidase (POD), and catalase (CAT), which detoxify the reactive oxygen species (ROS) produced under stress conditions.

### 4.2 Transcriptional regulation and hormonal signaling

These physiological responses are orchestrated at the genetic level by various transcription factors (TFs), acting as central hubs in stress-responsive signaling cascades. These regulatory networks are often influenced by hormonal signals, particularly abscisic acid (ABA), which can activate both ABA-dependent and ABA-independent pathways. Many studies elucidated critical roles of transcription factor families in regulating expression of stress-related genes in response to salinity. AP2/ERF family is central to regulating stomatal closure, antioxidant defenses, and osmotic adjustment. DREB TFs, for example, enhance salt tolerance by regulating osmoprotection, with some like *OsDREB1F* participating in ABA-dependent pathways while others function independently (Ito et al., 2006). A related TF, *OsEREBP1*, enhances salt tolerance through the jasmonic acid (JA) and ethylene pathways, further demonstrating the interconnectedness of hormone signaling (Wang et al., 2020). bZIP family is characterized by a conserved basic leucine zipper domain, these TFs are primarily involved in the ABA-dependent pathway. *OsABF2*, a key member, acts as a positive regulator of salt stress by binding to ABRE to activate downstream genes (Hossain et al., 2010), while *OsHBP1b* enhances antioxidant defenses (Das et al., 2019). The NAC family modulates other TFs like *DREB*, *MYB*, and *bZIP* to enhance salt tolerance by upregulating genes for osmoprotection, ion homeostasis, and antioxidant defense. Key members like *ONAC022* and *OsNAC10* positively regulate salt tolerance through ABA-mediated pathways (Hong et al., 2016; Jeong et al., 2010). In addition, *MYB* and *Zinc Finger (ZF)* TFs also play critical roles in salt stress tolerance. *OsMYBc* enhances salt tolerance by regulating the expression of the ion transporter *OsHKT1;1* (Xiao et al., 2022). Meanwhile, C2H2-type ZF TFs like *ZFP179* and *ZFP252* positively regulate the synthesis of compatible solutes like proline (Sun et al., 2010), while the *DST* gene, another C2H2-type ZF TF, acts as a negative regulator of salt tolerance by modulating stomatal closure (Huang et al., 2009).

Beyond these internal cellular and genetic mechanisms, the plant’s response is further shaped by its interaction with the soil environment. Recent research has revealed that the rhizosphere microbiome plays a significant role in mediating salt tolerance. A key discovery was the *SST* (Seedling Salt Tolerant) gene, whose mutation in rice leads to enhanced growth under salt stress by reducing Na^+^ uptake and increasing K^+^ accumulation (Lian et al., 2020). This improved ion homeostasis is coupled with a significant shift in the rhizosphere microbiome, suggesting a link between the plant’s internal genetics and its ability to recruit beneficial microbes. Specifically, salt-tolerant rice varieties maintain greater bacterial diversity in the rhizosphere compared to salt-sensitive ones, and they actively recruit distinct bacterial consortia. For example, tolerant rice plants enrich bacteria with functional genes related to saline-alkali tolerance, such as those for ABC transporters and biofilm formation (Lei et al., 2025). This biotic interaction underscores the need for a consortium approach using multiple beneficial microbes rather than a single strain to effectively enhance salt tolerance in agriculture. The combined evidence from molecular, physiological, and microbial studies presents a more holistic view of rice remarkable resilience to salt stress.

## 5 Roles of rice genotype and root exudates in shaping the rhizosphere microbiome

### 5.1 The role of root exudates and soil metabolites

The plant’s response to environmental stress is not limited to internal cellular and genetic mechanisms. It is further shaped by its interaction with the soil environment. Rice plant is not a passive recipient, instead, it actively influences the associated PGPR communities and their activities. This sophisticated, mutualistic relationship is driven by several factors, including the release of root exudates that act as chemical signals influencing PGPR activity and colonization. Recent research has revealed that the rhizosphere microbiome plays a significant role in mediating salt tolerance, a process strongly influenced by root exudates, soil metabolites, and the plant’s genotype (Yusuf et al., 2025; Fan et al., 2025). These factors collectively drive a sophisticated feedback loop that enhances plant growth and resilience. The rhizosphere is a dynamic interface where plants, microbes, and soil continuously interact, with root exudates serving as a crucial communication factor (Bacilio-Jiménez et al., 2003). These exudates, composed of small, low-molecular-weight organic compounds known as soil metabolites—including sugars, amino acids, and organic acids—serve as a primary source of carbon and nutrients for microorganisms (Song et al., 2024; Bolan et al., 2011). They also enable plants to selectively recruit beneficial microbes, such as PGPR (Parasar, Sharma, and Agarwala 2024). Rice root exudates induce a higher chemotactic response for endophytic bacteria, facilitating their colonization (Zhang et al., 2022). In addition to providing nutrients, these exudates contain secondary metabolites like phenolic compounds, flavonoids, and volatile organic compounds that act as signaling molecules, antimicrobial agents, or chemoattractants, directly influencing the structure and function of microbial communities (Bais et al., 2006; Hu et al., 2018; Zhalnina et al., 2018). Beyond plant-derived exudates, the soil metabolite pool is also shaped by microbial byproducts and specific signaling compounds like quorum-sensing molecules, which regulate microbial communication and impact plant-microbe interactions (Venturi and Fuqua, 2013). Quorum-sensing molecules, like N-acyl-homoserine lactones, regulate microbial communication, impacting plant-microbe interactions (Venturi and Fuqua, 2013). Precise identification and quantification of these metabolites are critical for understanding rhizosphere dynamics under environmental stresses. Gas Chromatography-Mass Spectrometry (GC-MS) and Liquid Chromatography-Mass Spectrometry (LC-MS) are standard for metabolite profiling. GC-MS detects volatile and semi-volatile compounds, such as VOCs, while LC-MS analyzes polar and non-volatile metabolites, including organic acids and amino acids (Lisec et al., 2006). These complementary platforms enable comprehensive analysis of the rhizosphere metabolome. Emerging techniques, such as Matrix-Assisted Laser Desorption/Ionization Imaging Mass Spectrometry (MALDI-IMS), provide spatial resolution of metabolite distribution around rice roots, revealing localized interactions between roots and microbes (Yang et al., 2012). This is particularly valuable for studying metabolite gradients in rice paddies. Complex datasets from untargeted metabolomics are analyzed using bioinformatics and statistical tools, such as principal component analysis (PCA) or partial least squares-discriminant analysis (PLS-DA), to identify key metabolites differentiating rice genotypes or stress conditions (Li et al., 2023).

### 5.2 Genotype-specific shaping of the rhizosphere community

Plant genotypes substantially impact the composition of its root-associated microbial communities, and this effect is the most pronounced in the rhizosphere (Edwards et al., 2015). Different rice genotypes possess varying root exudate profiles, which influence the recruitment of specific bacteria. For example, the abundance of Bacillus and Candidatus_Koribacter differed significantly between rice genotypes under drought conditions (Li et al., 2023). A recent study by Zhong et al. (2025) analyzed rhizosphere microbial communities in rice varieties Jida177 and Tongxi933 under saline-alkaline stress. Proteobacteria dominated high-stress soils, with Jida177 showing higher microbial diversity and better stress tolerance, reducing soil salinity by 73%. Microbial functions varied, with pH as the primary driver of community structure (Zhong et al., 2025). An important finding was related to the *SST* (Seedling Salt Tolerant) gene, whose mutation in rice led to enhanced growth and improved ion homeostasis under salt stress by reducing Na^+^ uptake and increasing K^+^ accumulation (Lian et al., 2020). Importantly, this genetic alteration was coupled with a significant shift in the rhizosphere microbiome, suggesting a direct link between the plant’s internal genetics and its ability to recruit beneficial microbes. Salt-tolerant rice varieties, in general, are found to maintain greater bacterial diversity in the rhizosphere and actively recruit distinct bacterial consortia. For instance, these tolerant plants enrich bacteria with functional genes related to saline-alkali tolerance, such as those for ABC transporters and biofilm formation (Lei et al., 2025). The mutual interaction between PGPR and rice is a sophisticated and cooperative relationship. PGPR enhances rice growth and stress resilience through various modulations, while the rice plant, in turn, influences the survival, activity, and community structure of these beneficial bacteria. This understanding underscores the need for a consortium approach using multiple beneficial microbes rather than a single strain to overcome the inherent limitations of using a single microbial strain in complex agricultural systems, especially under challenging environmental conditions.

## 6 Omics approaches in PGPR-mediated salt stress mitigation in rice

### 6.1 Physiological and morphological improvements

**Figure 2:**
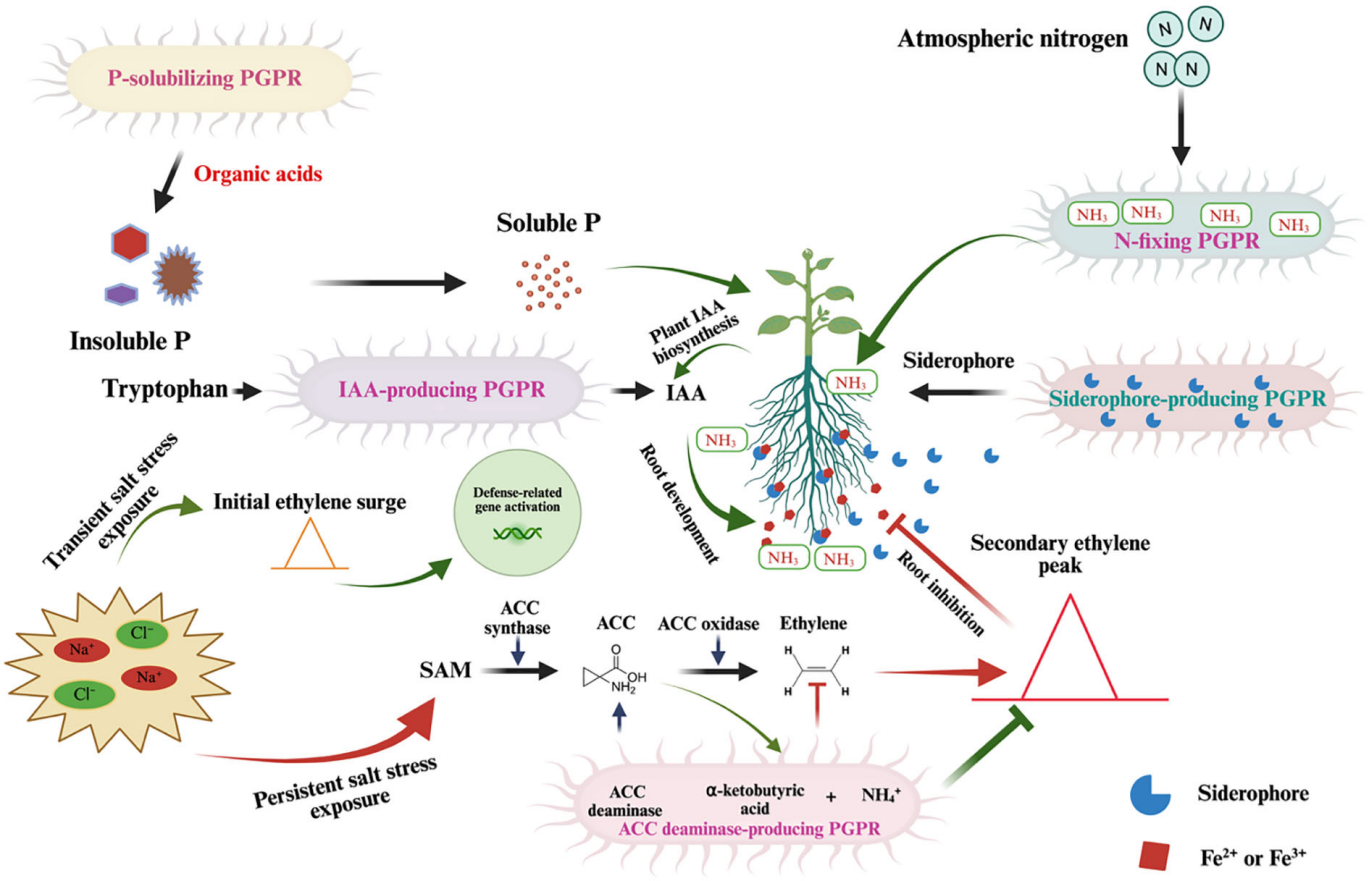
Mechanisms of PGPR in Enhancing Plant Resilience Under Salt Stress. This schematic diagram illustrates the multifaceted roles of PGPR in supporting plant growth under salt stress conditions. (Left Panel): Salt stress, characterized by elevated Na^+^ and Cl⁻ levels, triggers an ethylene surge via the S-adenosylmethionine (SAM) pathway, involving 1-aminocyclopropane-1-carboxylic acid (ACC) synthase and ACC oxidase. Persistent salt stress leads to an overproduction of ethylene, which inhibits root development, but PGPR mitigate this stress through ACC deaminase activity, reducing ethylene levels. PGPR enhance nutrient availability by solubilizing insoluble phosphorus (P) into soluble P via organic acid production. (Center Panel): PGPR facilitate IAA (indole-3-acetic acid) biosynthesis, promoting root development and nutrient uptake. (Right Panel): Nitrogen-fixing PGPR convert atmospheric nitrogen (N_2_) into ammonia (NH_3_), which is further assimilated by the plant. Additionally, siderophores produced by PGPR chelate Fe²^+^/Fe³^+^, improving iron availability.

Implementations of PGPR significantly improve rice plants physiologically and morphologically (**Figure 2**; **Table 2**). Inoculation with *Azospirillum brasilense* significantly improves total and root plant mass in rice plants grown under high salt concentrations (100 mM and 200 mM NaCl), with improvements observed seven and fourteen days after treatment (Degon et al., 2023). *Bacillus siamensis* BW-treated seeds exhibit rapid germination compared to untreated controls and show minimal reduction in shoot and root growth at the early seedling stage under saline conditions (75 mM to 150 mM NaCl) (Oubaha et al., 2024). Similarly, rice plants inoculated with *Glutamicibacter* sp. YD01 show less reduction in root and shoot length under NaCl stress, as this bacterium promotes root growth in seedlings. *Glutamicibacter* sp. YD01 also significantly enhances K^+^, reduces Na^+^ content in both leaves and roots, and increases relative water content (RWC) in rice seedlings (Ji et al., 2020). A study by Qin et al. (2018) found that *Glutamicibacter halophytocola* strain KLBMP 5180, isolated from the root tissue of *Limonium sinense*, significantly promoted host growth under NaCl stress by increasing concentrations of total chlorophyll, proline, antioxidative enzymes, flavonoids, K^+^, and Ca^2+^ in *L. sinense* leaves (Qin et al., 2018). *Enterobacter asburiae* D2 inoculation specifically promotes rice growth, including root length and dry weight, under both neutral (NaCl) and alkaline (Na_2_CO_3_) salt conditions (Ning et al., 2024). Additionally, *Pseudomonas promysalinigenes* RL-WG26 significantly increases plant biomass, root surface area, and root length in rice seedlings under both normal and saline conditions (Ren et al., 2024).

**Table 2:** Supportive effects of PGPR on rice plant under salt stress conditions.

Species	Salt tolerance testing conditions for PGPR	Plant growth promoting traits	Salt tolerance testing conditions for rice plants	Rice variety	Beneficial effects on plant hosts	References
*Bacillus amyloliquefaciens* RWL-1	120 and 250 mM NaCl	ABA, aspartic acid, glutamic acid, threonine, serine, glycine, methionine, alanine, valine, tyrosine, phenylalanine, isoleucine, lysine, arginine, and proline	120 and 250 mM NaCl	Japonica rice (*Oryza sativa* L. “Jin so mi”)	Increased shoot and root length, fresh and dry seedling weight, chlorophyll, endogenous salicylic acid	(Shahzad et al., 2017)
*Brevibacterium linens* RS16	1.7 M NaCl	H^+^-ATPase activity Hydroxyectoine biosynthesis gene (*ectD*) and hydroxyectoine accumulation in RS16 under salt stress conditions, N fixation Production of ACC deaminase, IAA, ammonia.	50 and 100 mM NaCl	Salt-tolerant (FL478) and salt-sensitive (IR29) rice cultivars (*Oryza sativa* L.)	Reduced lipid peroxidation, H_2_O_2_, lipoxygenase. Regulate H^+^ ATPase activity. Increased shoot and root length, dry mass, antioxidant enzyme, carotenoids, plant vacuolar H^+^ ATPase activities	(Chatterjee et al., 2018)
*Streptomyces* sp. *GMKU 336*	6% (w/v) NaCl	Phosphate solubilization, siderophore and ACC deaminase production	150 mM NaCl	Thai jasmine rice Khao Dok Mali 105 cultivar (*Oryza sativa* L. cv. KDML105)	Increased plant growth (shoot and root length, shoot and root fresh weight shoot and root dry weight), chlorophyll, proline, water content, K^+^, Ca^2+^. Decreased ethylene, ROS, Na^+^, Na^+^/K^+^ ratio. Downregulated genes *ACO1*, *EREBP1*, *MAPK5*. Upregulated genes *BADH1*, *NHX1*, *SOS1*, *Cam1-1*, *CuZn-SOD1*, *CATb*	(Jaemsaeng et al., 2018)
*Glutamicibacter* sp. YD01	5–10% (w/v) NaCl	ACC deaminase, IAA production	100 and 200 mmol/L	Not mentioned	Reduced Na^+^ accumulation, electrolyte leakage, ROS, ethylene, MDA, electrolyte leakage, ACC accumulation. Increased fresh and dry weight, chlorophyll, net photosynthesis rate, stomatal conductance, higher K^+^ levels, POX, SOD, GR. Upregulated genes *OsHKT1*, *OsNHX1*, *OsPOX1*, *OsFeSOD*, *OsGR2*, *OsWRKY1*, *OsDREB2A*	(Ji et al., 2020; Hussain et al., 2022)
*Bacillus tequilensis, Providencia stuartii and Bacillus aryabhattai*	2 M NaCl	IAA, exopolysaccharide production	EC of 8 dS/m	Salt-tolerant variety BRRI dhan67, moderate salt-tolerant variety Putra-1, and sult-susceptible variety MR297	Increased photosynthesis rate, transpiration, stomatal conductance, filled grain, 1000 grain weight, grains/plant	(Shultana et al., 2020a)
*Streptomyces albidoflavus* OsiLf-2	150 mmol/L NaCl	Intracellular proline, ectoine, biofilm, intracellular polysaccharides	150 mmol/L NaCl	*Oryza sativa* cv. indica 9311	Increased plant dry weight, survival rate of seedlings. Improved panicle length, 1000-grain weight, filled grains/panicle, effective tiler number, soluble sugar, chlorophyll, carotenoids, net photosynthetic rates. Increased SOD, CAT, POD, K^+^/Na^+^ ratio. Reduced MDA, REC, DAB, shoot and root Na^+^. Upregulated *OsALAD*, *OsPSY3*, *OsatpE*, *OsSOS1*, *OsNHX1*, *OsHKT1*, *OsLEA3*, *OsRab16A*, *OsDREB2A*	(Niu et al., 2022)
*Brevibacterium sediminis IBGE3C, BTCoSo2, BTCoR2*	12% (w/v) NaCl	Not mentioned	1% (w/v) NaCl	Salinity susceptible-variety BRRI dhan29 and salinity-tolerant variety BINAdhan-10	Increased shoot and root length, shoot and root dry weight	(Mahmud-Ur-Rahman et al., 2022)
*Streptomyces griseoincarnatus RB7AG*	10% (w/v) NaCl	Produce siderophores, IAA, ammonia, hydrogen cyanide (HCN)	6% (w/v) NaCl	Not mentioned	Increased shoot and root length, chlorophyll, proline, antioxidant enzymes (CAT, SOD, POD) Reduced lipid peroxidation	(Behera et al., 2023)
*Azospirillum brasilense* Sp245	Not mentioned	Not mentioned	100 and 200 mM NaCl	Nipponbare variety	Induced genes related to defense and stress response, ABA, JA, nutrient transport	(Degon et al., 2023)
*Bacillus* sp. PnD	Not mentioned	IAA, siderophore production phosphate solubilization	1% (w/v) NaCl	Amal-Mana variety	Increased total chlorophylls, carotenoids, protein, N, biomass, seed germination	(Dutta et al., 2023)
*Agrobacterium tumefaciens* (B1), *Bacillus subtilis* (B2), *Lysinibacillus fusiformis* (B3)	3.5%	Not mentioned	1 and 1.5% (w/v) NaCl	Binadhan-10 variety	Increased total length, fresh and dry weight, root length, shoot length, chlorophyll, germination rate	(Mahmud et al., 2023)
*Pseudomonas promysalinigenes* RL-WG26	Not mentioned	Tryptophan, IAA, betaine, ACC deaminase	1400 µS/cm and 1600 µS/cm	RS86 variety	Increased survival rate, fresh and dry weight, root surface area and length, chlorophyll, K^+^, Ca^2+^ levels, proline, CAT, POD, SOD, chlorophylls and carotenoids. Reduced lipid peroxidation, Na^+^, Cl^–^, Na^+^/K^+^ ratio	(Ren et al., 2024)

### 6.2 Gene regulation and molecular mechanisms

PGPR improve rice growth under salt stress by regulating the expression of key genes involved in defense and stress response (Giannelli et al., 2023). Transcriptomic studies have shown differentially expressed genes (DEGs) in *A. brasilense*-treated, salt-stressed rice roots, including those involved in defense and stress response (Degon et al., 2023). Other *Bacillus* strains, like NMTD17 and GBSW22, highly stimulated the expression of various DEGs related to salt stress, including *OsSAMDC2, OsDREB1F, OsEREBP2, OsLEA3-1, OsERF104*, and *OsCYP89G1* (Ali et al., 2022). *OsSAMDC2* is involved in polyamine biosynthesis, which helps mitigate oxidative damage under salinity (Sackey et al., 2025). Its expression is regulated by *OsNAC45*, a transcription factor involved in the salt stress response (Zhang et al., 2020). *OsEREBP2* functions downstream of *OsNAC45*, and its expression is upregulated in response to salt treatment, suggesting its involvement in salt stress signaling (Zhang et al., 2020). OsLEA3 is a late embryogenesis abundant group 3 protein and its accumulation in the vegetative tissues of transgenic rice helped improve tolerance to salt stress and water deficit (Hu, 2008). While *OsERF106* acts as a negative regulator of shoot growth and salinity tolerance in rice, the functions of its relative, *OsERF104*, in response to salinity remain unknown (Chen et al., 2021). *OsCYP89G1*, a CYP450 family member, encodes for cytochrome P450 monooxygenase. Its expression is upregulated by NaCl and repressed by ABA (Zhang et al., 2020). Although its specific role in rice salinity response is unclear, the overexpression of another family member, *OsCYP75B4*, improved salt tolerance in transgenic rice plants (Ruan et al., 2025). *OsDREB1F* belongs to the DREB transcription factor family, which plays a key role in plant stress signaling. Its expression is induced by salt, drought, cold stresses, and ABA, and its overexpression has been shown to enhance tolerance to these stresses in rice and Arabidopsis (Wang et al., 2008).

### 6.3 ROS, hormones, and ion transport

Salinity stress typically leads to oxidative stress and the accumulation of reactive oxygen species (ROS), which plants counteract using antioxidant enzymes like catalases (CAT), glutathione-S-transferases (GST), and superoxide dismutases (SOD) (Asif et al., 2023). Accordingly, genes encoding these enzymes are often upregulated in salt-stressed plants. However, PGPR treatment can have varied effects on these genes. *A. brasilense* treatment suppressed most of these antioxidant genes in salt-stressed rice roots, suggesting that *A. brasilense* inoculation relieves stress and reduces the need for the plant’s own antioxidant response (Degon et al., 2023). Similarly, *Brevibacterium linens* RS16 reduced plant antioxidant enzyme activity and lipid peroxidation (Chatterjee et al., 2018). Conversely, other PGPR strains, such as *Glutamicibacter* sp. YD01, *Bacillus siamensis* BW, *Pseudomonas promysalinigenes* RL-WG26, and *Microbacterium ginsengiterrae* S4, significantly increased the activity of antioxidant enzymes (SOD, Peroxidase, CAT, Ascorbate Peroxidase, and Glutathione Reducer) in rice seedlings under salinity stress (Oubaha et al., 2024; Sarkar et al., 2018; Ren et al., 2024; Chinachanta et al., 2023; Khan et al., 2021; Ji et al., 2024). This response is often accompanied by the upregulation of genes encoding these enzymes, including *OsPOX1*, *OsFeSOD*, *OsGR2*, *OsCATA*, and *OsAPX1* (Asif et al., 2023; Sarkar et al., 2018; Khan et al., 2021). PGPR treatment also alters the expression of salt-induced ABA and JA signaling genes in rice roots (Degon et al., 2023). Other studies using *Bacillus* spp., *Lysinibacillus fusiformis*, *Lysinibacillus* sp*haericus*, and *Brevibacterium pityocampae* also reported decreased ABA content in PGPR-inoculated rice plants under salt stress (Khan et al., 2021; Asif et al., 2023), implying that these PGPRs alleviate stress to the extent that these hormone pathways are not strongly activated. ACC deaminase-producing PGPRs, such as *Glutamicibacter* sp. YD01 and *P. promysalinigenes* RL-WG26, reduce ACC content and ethylene production. This, in turn, downregulates ethylene-responsive genes (e.g., *OsERF1*) and alleviates growth inhibition caused by excessive ethylene under stress (Ganie et al., 2022; Ji et al., 2020; Ren et al., 2024). PGPRs also modulate genes involved in Na^+^ and P transport and Ca^2+^ signaling. Key genes like *OsNHX1* (Na^+^/H^+^ antiporter), *SOS1* (Salt Overly Sensitive 1), and *OsHKT1* (high-affinity K^+^ transporter) are strongly regulated (Degon et al., 2023; Ganie et al., 2022). *Glutamicibacter* sp. YD01 upregulates *OsHKT1* expression (Ganie et al., 2022), while *Brevibacterium linens* RS16 induces vacuolar H^+^ ATPase activity (Chatterjee et al., 2018), which helps to remove Na^+^ from the cytosol or maintaining a low Na^+^ concentration in plant cells. *Bacillus* spp. upregulates *OsPIN1A* expression (Khan et al., 2021), which is involved in auxin efflux and root development, thereby improving nutrient acquisition under salt stress (Xu et al., 2005).

### 6.4 Transcription factors and proteins

Regarding TFs and other stress-related proteins, PGPRs influence the expression of various TF families, including *WRKY*, *DREB, ERF, MYB*, and *bZIP*, which are crucial regulators of plant stress responses (Ali et al., 2022). *Glutamicibacter* sp. YD01 upregulates *OsWRKY11* and *OsDREB2A* (Ji et al., 2020), and *Microbacterium ginsengiterrae* S4 induces the upregulation of *OsWRKY76* (Ji et al., 2024). Genes associated with abiotic stress tolerance, such as *OsLEA3* and *OsRab16A*, also show markedly higher expression levels in *Streptomyces albidoflavus* OsiLf-2-inoculated rice under salt conditions (Niu et al., 2022; Ganguly et al., 2012; Ganguly et al., 2020). PGPR inoculation also leads to the upregulation of chaperone proteins, like the 60 kDa chaperonin, HSP20, GroEL, and calreticulin, which are crucial for refolding denatured proteins and maintaining protein homeostasis under stress (Meng et al., 2024). Some defense-related genes like chitinases also show altered expression patterns with *A. brasilense* inoculation (Degon et al., 2023).

### 6.5 Exopolysaccharides as a physical barrier

EPS produced by halotolerant PGPR (e.g., *Bacillus cereus* DB2, *Bacillus tequilensis*, *Bacillus siamensis* BW, *Enterobacter* sp. JIV1) can bind Na^+^ ions in the rhizosphere, making them less available for plant uptake (Debapriya Choudhury et al., 2024; Oubaha et al., 2024; Shultana et al., 2020b; Wang et al., 2022; Ning et al., 2024; Ji et al., 2024). This physical barrier reduces the ionic stress on plant roots, thereby influencing the plant’s gene expression response to salinity.

## 7 Studies of PGPR on rice in Vietnam-advances and limitations

### 7.1 Advances in PGPR studies in Vietnam

Studies on PGPR on rice have shown promising results in Vietnam (**Table 3**). For example, a significant field study across 20 farms over four consecutive growing seasons evaluated BioGro 2, a commercial biofertilizer containing *Pseudomonas fluorescens, Bacillus subtilis, Bacillus amyloliquefaciens*, and *Candida tropicalis* (Nguyen et al., 2017). While the product successfully replaced 23–52% of N requirements, maintaining grain yields comparable to conventional chemical fertilizers, its inability to substitute for P and K demands represents a significant limitation. Furthermore, the wide range of effectiveness (23–52%) and the notable influence of timing and dosage on its performance highlight a critical need for optimized, context-specific application strategies to ensure consistent results and minimize risks for farmers. In response to increasing saline intrusion in Vietnam’s coastal regions, such as Soc Trang and Ben Tre, researchers have focused on isolating salt-tolerant PGPR. A study in 2018 obtained 48 salt-tolerant isolates, of which 22 produced indole-3-acetic acid (IAA) and 17 showed potential for N fixation and PO_4_^3^⁻ solubilization (Cuong et al., 2020). Six of these isolates, from the genera *Bacillus*, *Halobacillus*, *Aeromonas*, and *Klebsiella*, exhibited all three traits, indicating their potential as multifunctional biofertilizers for saline-affected soils.

**Table 3:** Effects of PGPR on growth parameters, grain yield of some rice varieties and physio-chemical properties of soils.

Bacterial trains	Plant-promoting traits	Field/Experiment conditions	Beneficial effects on rice plants	Agronomic benefits in the field	References
*Burkholderia vietnamiensis* TVV75	N fixation IAA and siderophore production	- Hoc Mon District (acid sulfate soil); rice variety OM5971. - Nha Be District (saline acid sulfate soil); local rice variety Nang Huong. - Binh Chanh District (slightly acid sulfate alluvial soil), local rice variety Nang Thom.	Increased number of panicles per pot, shoot height (13%), shoot weight (33%), root weight (57%), leaf surface (8%), 1000-grain weight, filled grain/pot, and grain yield (20% in Nha Be, 22% in Hoc Mon, 13% in Binh Chanh)	Not mentioned	(Trân Van et al., 2000)
*Ochrobactrum ciceri* TCM_39, *Microbacterium* *neimengense* MCM_15, *Klebsiella aerogenes* LCT_01, *Olivibacter jilunii* PTST_30 và *Citrobacter freundii* RTTV_12	Si and P solubilization IAA production	Rice seedlings were grown in test tubes under laboratory conditions Hoagland + 0.3% NaCl Rice variety LP5	Increased germination rate, plant height, root length, root number, total biomass	Not mentioned	(Đường et al., 2018)
*Burkholderia* sp. PL9 *Acinetobacter* sp. GH1-1	N fixation IAA production	Saline soil in rice-shrimp farming system, Soc Trang Province Salt-tolerant rice variety LP5	Increased plant height, tillers/m^2^, panicles/m^2^, panicle length, filled grains/panicle, 1000-grain weight, yield Decreased unfilled grains	Reduced 50% N fertilizer use in rice production	(Huy and Hiệp, 2019)
*Rhodopseudomonas palustris* strains (TLS06, VNW02, VNW64 and VNS89)	P solubilization	ASS collected from Phung Hiep District, Hau Giang Province and Hon Dat District, Kien Giang Province ASS-tolerant rice variety OM5451	Increased plant height, number of panicles, panicle length, total spikelet/panicle, and yield.	Increased phosphatase activity, pH Decreased Al^3+^, Fe^2+^ in soil	(Khuong et al., 2018)
Biofertilizers containing four acid-resistant *Rhodopseudomonas palustris* VNW64, VNS89, TLS06 and VNS02	Reduce Al^3+^ and Fe^2+^ toxicity in acid sulfate soil (ASS) IAA, siderophore and 5-Aminolevulinic acid (ALA) production EPS secretion	ASS collected from Phung Hiep district, Hau Giang province under net house ASS-tolerant rice variety OM5451	Increased N level in rice stem Reduced Al and Fe uptake in rice plants	Immobilization of Al^3+^ and Fe^2+^ in acid sulfate soil	(Xuân et al., 2019)
*Ochrobactrum ciceri* TCM_39, *Microbacterium* *neimengense* MCM_15, *Klebsiella aerogenes* LCT_01, *Olivibacter jilunii* PTST_30 and *Citrobacter freundii* RTTV_12	Si and P solubilization IAA production	Saline soil in rice-shrimp farming system, Bac Lieu Province Salt-tolerant rice variety Mot Bui Do	Increased soluble Si level in dry biomass, chlorophyll, and yield Enhanced stem internode stiffness	Increased soluble Si level in soil	(Đường and Nghĩa, 2020)
Salt-tolerant microbial formulation NPISi: *Bacillus aquimaris* KG6-3, *Burkholderia* sp. BL1-10, *Bacillus megaterium* ST2-9, and *Citrobacter freundii* RTTV_12	N fixation Si and P solubilization IAA production	Saline soil in rice-shrimp farming system, Bac Lieu Province Salt-tolerant rice variety Mot Bui Do	Increased plant height, tiller/m^2^, Si level in rice stem, yield	Increased soluble Si level in soil Increased NH_4_^+^, NO_3_^-^, soluble P and K, beneficial bacteria densities in soil	(Nguyễn et al., 2021)
*Pantoea* sp. X4.1 *Bacillus subtilis* X8.2	N fixation	Rice seedlings were grown in test tubes under laboratory conditions Rice variety OM 4218	Increased root and stem length, dry biomass of 15-day-old seedlings	Not mentioned	(Giang et al., 2024)

Purple nonsulfur bacteria (PNSB), like *Rhodopseudomonas palustris* and *Rhodopseudomonas harwoodiae*, have also been explored as biofertilizers and bioremediators. Studies have shown they can enhance rice growth and grain yield while improving soil fertility and reducing toxic Mn accumulation (Khuong et al., 2017; 2022). Field trials combining mixed PNSB with 75% of the recommended NP fertilizer achieved yields comparable to those obtained with 100% NP fertilizer. While these results are promising, the reproducibility of these findings across Vietnam’s diverse acid sulfate soils has not been comprehensively evaluated. The effectiveness of these formulations may vary significantly with regional soil chemistry and climatic conditions. Similarly, other studies have investigated the growth-promoting effects of silicate-solubilizing bacteria (SSB) and N-fixing strains like *Sinorhizobium fredii* and *Azospirillum* spp (Điệp, 2005; Chiêu and Hiệp, 2010; Đường et al., 2018). These studies have provided evidence that such microbes can improve nutrient uptake, reduce fertilizer dependency, and increase stress tolerance. The commercial microbial formulation NPISi was shown to significantly increase grain yield and soil health in a rice-shrimp farming model (Nguyễn et al., 2021). However, a notable observation that the formulation elevated K levels in rice despite the absence of K-solubilizing bacteria highlights a critical knowledge gap regarding the full range of microbial functions and their complex interactions in the soil.

### 7.2 Limitations in existing studies and further research directions

In summary, previous studies had some limitations as many field experiment were conducted for only one cropping season (Minh et al., 2020; Nguyễn et al., 2021). This drawback raises concerns reproducibility across seasons and long-term impacts of soil improvement products in salt-affected areas. Although the focus of these studies on specific soil conditions, e.g., salt-affected soil in a rice-shrimp farming system, salt-affected clay loam soil, and acid sulfate soil, is understandable due to limitations of time and budgets, their specificity may limit the generalizability of these findings (Đường and Nghĩa, 2020; Huy and Hiệp, 2019; Xuân et al., 2019). Therefore, future research should be systematically conducted across multiple-season field trials and on a wider range of soil types to thoroughly evaluate long-term effectiveness, consistency and applicability of these PGPR-based approaches. All studies provided initial characterization of the bacterial strains (Đường et al., 2018; Đường and Nghĩa, 2020; Giang et al., 2024; Huy and Hiệp, 2019; Minh et al., 2020; Nguyễn et al., 2021; Xuân et al., 2019). However, these studies did not provide data regarding the long-term consistency of these bacteria over generations. Moreover, potential genetic drift and phenotypic changes over time and in large-scale production were not mentioned. Therefore, comprehensive studies on the genetics and beneficial traits’ stability over successive generations and production cycles are critical to ensure their consistent performance and reliability as biofertilizers for widespread applications. Additionally, studies on the shelf-life and long-term storage stability of these biofertilizer formulations under various conditions and extended periods should be extensively carried out.

## 8 Advances and challenges in salt-tolerant rice breeding in the Mekong Delta

Rice production in the Vietnamese Mekong Delta (VMD) faces mounting challenges due to salinity intrusion, climate variability, and infrastructural constraints. Breeding programs have responded with the development of salt-tolerant rice varieties (STRVs), yet limitations persist. Trade-offs in varietal traits—such as long growth duration, low yield potential, and poor grain quality—remain common among traditional and some modern STRVs like IR42 and Doc Phung (Quan and Lien, 2023; Tin et al., 2021). Additionally, hydrological models often fail to capture dynamic changes in irrigation infrastructure and upstream dam operations, complicating risk assessments (Wassmann et al., 2019). Farmer adoption is further hindered by short warning times, limited seed availability, and unequal access to information, particularly among women and ethnic minorities (Paik et al., 2020). Despite these constraints, breeding institutions such as the High Agricultural Technology Research Institute (HATRI) (Lang et al., 2020) and the Consortium for Unfavorable Rice Environments (CURE) (Paik et al., 2020) have made notable progress. HATRI has developed salt-tolerant varieties like HATRI 190, HATRI 192, and HATRI 170 using molecular markers and extensive field trials. Multi-location testing confirmed high survival rates and yield stability under salinity levels of 10–12 dS m^−1^. Grain quality traits—such as aroma and amylose content—were prioritized, and farmer participation through participatory variety selection (PVS) ensured relevance and adoption across salinity-affected provinces. CURE-related varieties, while less marketable, offer a low-cost insurance option against severe yield losses in unprotected high-salinity zones. Complementing institutional efforts, recent studies have identified promising genetic resources among Vietnamese landraces and crop wild relatives (CWRs). Most rice cultivars tolerate salinity up to 3 dS m^−1^, with yield reductions of 10% at 3.5 dS m^−1^ and 50% at 7.5 dS m^−1^ (Quan & Vo, 2017). Among 41 landraces evaluated by Anh et al. (2016), 15 showed moderate to high salinity tolerance, with varieties like Chanh Trui, Cuom Dang 2, and Nep Cuc performing comparably to Pokkali (Anh et al., 2016). These landraces survived up to 25–26 days under extreme salinity (16 dS m^−1^) and carried tolerance alleles linked to QTLs on chromosome 4 (RM217 marker). Five traditional VMD varieties—Nang Cha Rau, Nep Than, Trang Lun, Gie Hanh, and Nang Tich—exhibited tolerance across all growth stages at 9.38 dS m^−1^. Additionally, four CWR-derived lines (L180-3, L93-3, L71-3, and L33-6) yielded over 6.5 tons ha^−1^ under saline conditions, with early maturity and short plant height (Tin et al., 2021).

Marker-assisted selection (MAS) has facilitated the introgression of Saltol QTL into elite Vietnamese varieties such as AS996 and BT7 (Huyen et al., 2012; Linh et al., 2012). OM5451, a salt-tolerant variety developed through MAS, has become one of the most widely planted cultivars in the VMD, covering approximately 645,000 hectares across diverse soil types—including acid sulfate lands—in provinces like Long An, An Giang, Tien Giang, Đồng Tháp, Tra Vinh, Soc Trang, Bac Lieu, and Ben Tre. Moving forward, improving grain quality and market value of STRVs is essential to enhance their desirability and adoption. Long-term studies should evaluate varietal performance under varying salinity levels across multiple seasons. Successful examples like CTUSM1—a mutant cultivar with high amylose and protein content—demonstrate the feasibility of integrating quality traits into salt-tolerant lines (Quan & Vo, 2017).

For accurate warnings and predictions of salinity intrusion, risk maps need to be continuously updated to correctly reflect ongoing changes in land use and irrigation systems. Projections of sea-level rise, precipitation data, and impact from upstream dams should be incorporated to provide more comprehensive risk assessment. Given the significant neighborhood effects on farmer’s adoption, communication approaches should focus on the community and village levels rather than individuals. Farmers should be active contributors to rice variety development by actively involved in participatory variety selection (PVS) approach, addressing their preferences and specific needs (Tin et al., 2021). Together, these efforts offer a promising path toward sustainable rice production in the Mekong Delta’s increasingly saline landscape.

## 9 Conclusion

The agricultural system in the VMD faces mounting challenges due to both natural pressures—such as salinity intrusion, land subsidence, and water scarcity—and anthropogenic activities including upstream damming and unsustainable groundwater extraction. While major research institutes like the International Rice Research Institute (IRRI) and the Cuu Long Delta Rice Research Institute (CLRRI) have made significant strides in developing salt-tolerant rice varieties (STRVs), these genetic solutions alone are insufficient to ensure long-term resilience. Complementary strategies, particularly the application of indigenous PGPR, offer promising avenues for enhancing rice tolerance to abiotic stress. As demonstrated by multiple studies conducted in Vietnam, locally adapted PGPR strains exhibit strong compatibility with native soil conditions, climate, and rice genotypes—making them ideal candidates for biofertilizer development tailored to the VMD. However, several limitations persist. Advanced technologies such as omics-based approaches remain underutilized in Vietnamese rice research, restricting deeper insights into the molecular and metabolic mechanisms of salt tolerance. Additionally, many valuable studies by Vietnamese scientists are published in local-language journals, limiting their visibility and integration into global scientific discourse. These drawbacks must be urgently addressed to enable farmers in the VMD to improve their livelihoods through the adoption of high-quality salt-tolerant rice varieties. Furthermore, cutting-edge technologies should be integrated into future PGPR research to uncover the underlying mechanisms of rice–microbe interactions under salinity stress in this critical region.
